# Prion Protein Misfolding Affects Calcium Homeostasis and Sensitizes Cells to Endoplasmic Reticulum Stress

**DOI:** 10.1371/journal.pone.0015658

**Published:** 2010-12-29

**Authors:** Mauricio Torres, Karen Castillo, Ricardo Armisén, Andrés Stutzin, Claudio Soto, Claudio Hetz

**Affiliations:** 1 Center for Molecular Studies of the Cell, Institute of Biomedical Sciences, Faculty of Medicine, University of Chile, Santiago, Chile; 2 Biomedical Neuroscience Institute, Faculty of Medicine, University of Chile, Santiago, Chile; 3 Mitchell Center for Alzheimer's Disease and Related Brain Disorders, Department of Neurology, University of Texas Houston Medical School, Houston, Texas, United States of America; 4 Neurounion Biomedical Foundation, Santiago, Chile; 5 Harvard School of Public Health, Boston, Massachusetts, United States of America; Biological Research Center of the Hungarian Academy of Sciences, Hungary

## Abstract

Prion-related disorders (PrDs) are fatal neurodegenerative disorders characterized by progressive neuronal impairment as well as the accumulation of an abnormally folded and protease resistant form of the cellular prion protein, termed PrP^RES^. Altered endoplasmic reticulum (ER) homeostasis is associated with the occurrence of neurodegeneration in sporadic, infectious and familial forms of PrDs. The ER operates as a major intracellular calcium store, playing a crucial role in pathological events related to neuronal dysfunction and death. Here we investigated the possible impact of PrP misfolding on ER calcium homeostasis in infectious and familial models of PrDs. Neuro2A cells chronically infected with scrapie prions showed decreased ER-calcium content that correlated with a stronger upregulation of UPR-inducible chaperones, and a higher sensitivity to ER stress-induced cell death. Overexpression of the calcium pump SERCA stimulated calcium release and increased the neurotoxicity observed after exposure of cells to brain-derived infectious PrP^RES^. Furthermore, expression of PrP mutants that cause hereditary Creutzfeldt-Jakob disease or fatal familial insomnia led to accumulation of PrP^RES^ and their partial retention at the ER, associated with a drastic decrease of ER calcium content and higher susceptibility to ER stress. Finally, similar results were observed when a transmembrane form of PrP was expressed, which is proposed as a neurotoxic intermediate. Our results suggest that alterations in calcium homeostasis and increased susceptibility to ER stress are common pathological features of both infectious and familial PrD models.

## Introduction

Most neurodegenerative disorders, including amyotrophic lateral sclerosis, Alzheimer's, Parkinson's, Huntington's disease, and Prion-related disorders (PrDs), share common pathology features, highlighted by the accumulation of abnormal protein aggregates containing disease-specific misfolded proteins [1]. PrDs, also known as transmissible spongiform encephalopathies, are fatal neurodegenerative diseases affecting humans and other animals. Primary symptoms include rapid and progressive dementia, and ataxia [2]. Prion diseases are characterized by the spongiform degeneration of the brain accompanied by the accumulation of a misfolded and protease-resistant form of the cellular prion protein (PrP^C^), termed PrP^RES^ [2], [3]. The etiology of PrDs can be divided into three categories including hereditary, sporadic and infectious forms. Familial prion diseases, including Creutzfeldt-Jakob disease (CJD), fatal familial insomnia (FFI), and Gerstmann-Sträussler-Scheinker syndrome (GSS), are all linked to mutations in the gene encoding PrP^C^, *PRNP,* where at least 20 different mutations which trigger PrP misfolding and the generation of different levels and conformers of PrP^RES^ [2]. Infectious PrDs have an unusual mechanism of transmission and include *scrapie* in goat and sheep, chronic wasting disease in elk and deer, and bovine spongiform encephalopathy in cattle. The “protein-only” hypothesis postulates that infectious prion pathogenicity results from a conformational change of natively folded PrP^C^ from its primarily α-helical structure to an insoluble β sheet conformation, initiated by a direct interaction with PrP^RES^ present in the infectious agent. Then, PrP misfolding replicates in a cyclic manner where newly generated PrP^RES^ catalyzes the generation of more pathological prions at the expense of endogenous PrP^C^ [2], [4].

Like other secretory proteins, PrP^C^ undergoes extensive post-translational processing in the endoplasmic reticulum (ER) and Golgi [5]. After trafficking through the secretory pathway, fully matured PrP^C^ localizes to cholesterol-rich lipid rafts, and cycles through the endocytic pathway (review in [5]). During the folding process at the ER, around 10% of PrP^C^ is naturally misfolded and eliminated by the proteasome through the ER-associated degradation (ERAD) pathway [6]. The rate of ERAD-mediated degradation is substantially increased for familial PrP mutant forms [7], [8], [9], [10], [11]. Upon synthesis, most familial mutant PrP variants are retained and aggregated in the ER and Golgi, where they may exert their pathological effects (review in [12]). For instance, the neurotoxic mutants PrP^D178N/Met129^, linked to FFI, and PrP^PG14^ (nine-octapeptide insertion), linked to CJD, are partially retained in their transit through the secretory pathway [13]. The mutant PrP^Q217R^ linked to GSS is also retained at the ER and strongly interacts with the ER chaperone BiP/Grp78 [7], [14]. In addition, the experimental point mutation PrP^L9R/3AV^, leads to expression of an abnormal form of PrP called PrP^CTM^, exclusively located at the ER/Golgi as a transmembrane protein [9], [15], [16], [17]. PrP^CTM^ is proposed to be an intermediate species in PrP^RES^ formation, mediating prion neurotoxicity. In contrast to familial PrDs, the generation of infectious PrP^RES^ is proposed to occur at the plasma membrane and during its cycling through the endocytic pathway [18], [19], [20]. However, many studies in infectious PrDs models have shown the trafficking and accumulation of PrP^RES^ at the ER and cytosol [21], [22], [23], [24], [25], [26], [27].

Although the mechanism of PrP^RES^ pathogenesis is still highly controversial, accumulating data suggests that perturbations in ER homeostasis may contribute to neurodegeneration in PrDs. ER stress is triggered by a number of conditions that interfere with oxidative protein folding processes in the ER which lead to accumulation of intralumenal misfolded proteins (reviewed in [28]). The unfolded protein response (UPR) is an integrated signal transduction pathway activated by ER stress that transduces information about the protein folding status in its lumen to the cytosol and nucleus to increase protein folding capacity and decrease unfolded protein load [28]. Conversely, under chronic ER stress cells undergo apoptosis [29]. Chronic ER stress is associated with the pathogenicity of several neurodegenerative diseases linked to protein misfolding [1]. Upregulation of UPR-responsive chaperones, such as Grp78/BiP, Grp94, and Grp58/ERp57, and other ER stress markers is observed in the brain of patients affected with sporadic and new variant CJD [30], [31] and in different mouse models of scrapie [22], [30], [32], [33], [34], [35]. Besides, a recent report suggests that the expression of a GSS-linked PrP mutant triggers ER stress in a cellular model [36]. In addition, ER stress conditions induce PrP^C^ aggregation in neuronal cultures [29], [37] and in yeast models of PrD [38]. *In vitro* studies revealed that PrP^RES^ purified from the brain of scrapie-infected mice [30] or synthetic PrP-derived peptides induces ER stress [39], [40]. However, a direct link between PrP^RES^ and perturbations of ER homeostasis is still missing.

Different conditions alter the protein folding process at the ER lumen. Among them, sustained calcium release from the ER has been shown to affect the normal function of different ER-resident chaperones (review in [41], [42]). Suboptimal activity of ER chaperones triggers stress due to deficiency of protein folding, activating the UPR [43]. This mechanism has been recently proposed to operate in diseases such as lysosomal storage disorders and diabetes [44], [45]. It has been suggested that brain-derived infectious PrP^RES^ [30] and synthetic PrP peptides may affect calcium homeostasis [46], [47], [48], [49], [50], [51], but its possible contribution to PrP^RES^ neurotoxicity and ER stress has not been addressed directly. Here we have evaluated the possible impact of PrP^RES^ on ER stress responses and calcium homeostasis using both acute and chronic models of infectious PrDs, in addition to two familial PrDs models. Our data suggest that alteration of ER calcium homeostasis is a common and key neuropathological event in different models of PrDs, playing an important role in neurodegeneration.

## Results

### Cells chronically infected with RML scrapie prions are more susceptible to ER stress

**Figure 1:**
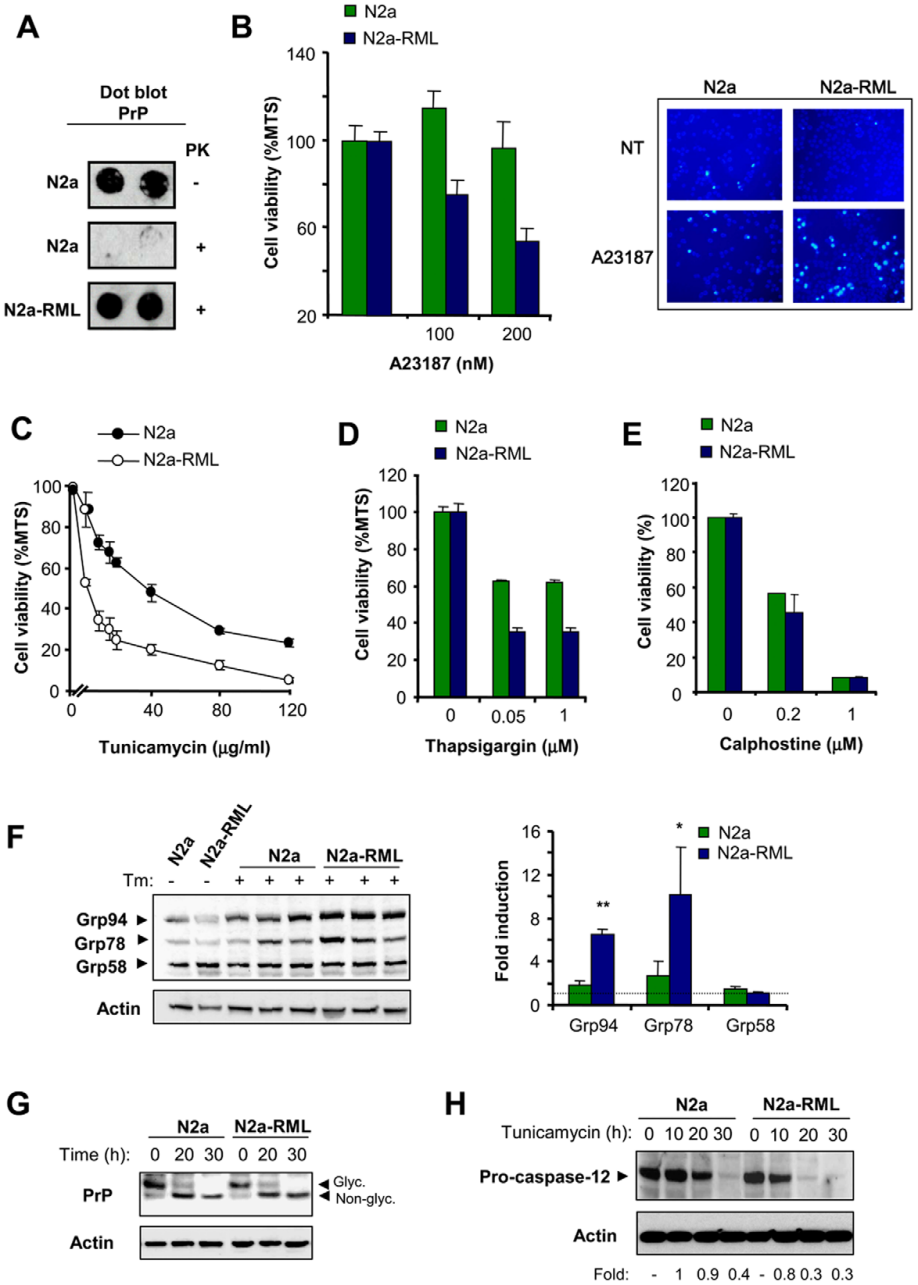
PrP^RES^ replication sensitizes Neuro2a cells to ER stress-mediated cell death. (**A**) Neuro2a cells were infected with RML-scrapie brain homogenate (N2a-RML) or uninfected (N2a). Prion replication in stable cultures was determined by PK treatment and dot blot analysis after 2 weeks of infection. (**B**) Left panel: N2a-RML and control cells were treated with different concentrations of A23187 for 48 h and cell viability was determined by MTS assay. Right panel: in parallel, Neuro2a cells were treated with 90 nM of the Ca^2+^ ionophore A23187, and after 48 h incubation apoptotic nuclear morphology was visualized after Hoechst dye staining. Infected and control cells were also subjected to treatments with indicated concentrations of tunicamycin (**C**), or thapsigargin (**D**), and after 48 h cell viability was quantified with the MTS assay. In panels B–D the mean and standard deviation from three independent experiments is shown. (**E**) As controls, cells were treated with different concentrations of calphostine for 48 h, and cell viability was quantified by MTS analysis. (**F**) N2a-RML and control cells were treated with 20 µg/ml tunicamycin for 30 h, and the expression levels of Grp58, Grp78, and Grp94 were analyzed by Western blot. Three different independent treatments are presented. Actin levels were determined as loading control. Right panel: Quantification of the relative induction levels is presented and normalized with the value obtained in non-treated cells. Mean and standard deviation is presented. Student t-test was used to analyze statistical significance (** *p*<0.001, * *p*<0.05). (**G**) N2a-RML and control cells were treated with 20 µg/ml tunicamycin for indicated time points, and the expression levels of PrP were analyzed by Western blot. Non- glycosylated and glycosylated PrP forms are indicated. (**H**) N2a-RML cells and control N2a cells were treated with tunicamycin and the levels of pro-caspase-12 processing were determined by Western blot. As controls, the levels of actin are shown. The pro-caspase-12 expression levels were quantified and the ratio between the expression levels of treated and control Cells is presented at the bottom of the gels.

To study the impact of prion replication in the physiology of the ER, we stably infected Neuro2a cells with the Rocky Mountain Laboratory (RML) scrapie strain, (here termed N2a-RML). To avoid selection effects, a clonal line was derived from Neuro2A to perform all experiments on an isogenic background. PrP^RES^ replication at expenses of endogenous PrP^C^ was stable for several weeks in culture (>8 weeks) as confirmed by dot blot detection of proteinase K (PK)-resistant PrP (Figure 1A). To test the effect of PrP^RES^ replication on the susceptibility of cells to ER stress, we treated control and N2a-RML cells with the ER stress-inducing agent A23187 (a calcium ionophore) and then monitored cell viability with two different assays. Although the infection of cells with RML scrapie prions did not induce evident spontaneous cell death of cell cultures (not shown), the exposure of N2a-RML cells to nanomolar concentrations of A23187 led to enhanced cell death when compared to non-infected cells (Figure 1B, left panel). Analysis of nuclear morphology after Hoechst staining confirmed the appearance of apoptotic nuclei in cells treated with A23187, which was enhanced in N2a-RML cells (Figure 1B, right panel). To confirm these results, we induced ER stress with tunicamycin (a N-linked glycosylation inhibitor) or thapsigargin (an inhibitor of ER-calcium ATPase, SERCA). Treatment of cells with both drugs lead to enhanced susceptibility to ER stress in N2a-RML cells when compared to non-infected cells (Figure 1C and D). Similar results were obtained when a different clone of Neuro2A was infected with RML prions and the phenotypes were not affected by cell passages (not shown). These effects were not observed when calphostine (Figure 1E) or staurosporine (not shown) were used to trigger intrinsic-cell death programs that are independent of ER stress. As an additional control, we selected a Neuro2a clone that was resistant to replicate scrapie prions (Neuro2a-RML-Ins). We exposed Neuro2a-RML-Ins cells to RML prions and then assessed their susceptibility to ER stress. Comparison of this cell line with N2a-RML cells confirmed the requirement of sustained prion replication to enhance the rate of ER stress-mediated cell death (Figure S1A).

To explore further the impact of prion replication on the susceptibility to ER stress, we monitored the levels of UPR activation in N2a-RML and control cells after exposure to tunicamycin. Analysis of Grp94, Grp78/BiP and Grp58/ERp57 levels revealed a stronger ER stress response in N2a-RML cells over time when compared to non-infected cells, manifested by a marked increase in the expression of these three ER stress-responsive chaperones (Figure 1F). No differential effects on total levels of PrP or its de-glycosylation upon tunicamycin treatment were observed in N2a-RML when compared to control cells, indicating that this compound was equally effective in both cell cultures (Figure 1G). We also monitored pro-caspase-12 processing, another marker of ER stress [52]. We observed an enhancement in the rate of pro-caspase-12 processing in N2a-RML cells undergoing ER stress compared to non-infected cells (Figure 1H). We were not able to detect the active fragments of caspase-12 in Neuro2a using three different antibodies, suggesting that the protein is unstable. To validate the functional role of caspase-12 in our experimental system, we expressed a dominant negative form of caspase-12 in Neuro2a cells and then assessed their susceptibility to ER stress (Figure S1B). Taken together, our results indicate that prion replication sensitized Neuro2a cells to ER-related injuries, suggesting that PrP^RES^ disturbs the homeostasis of this organelle.

### Prion replication affects ER calcium homeostasis

**Figure 2:**
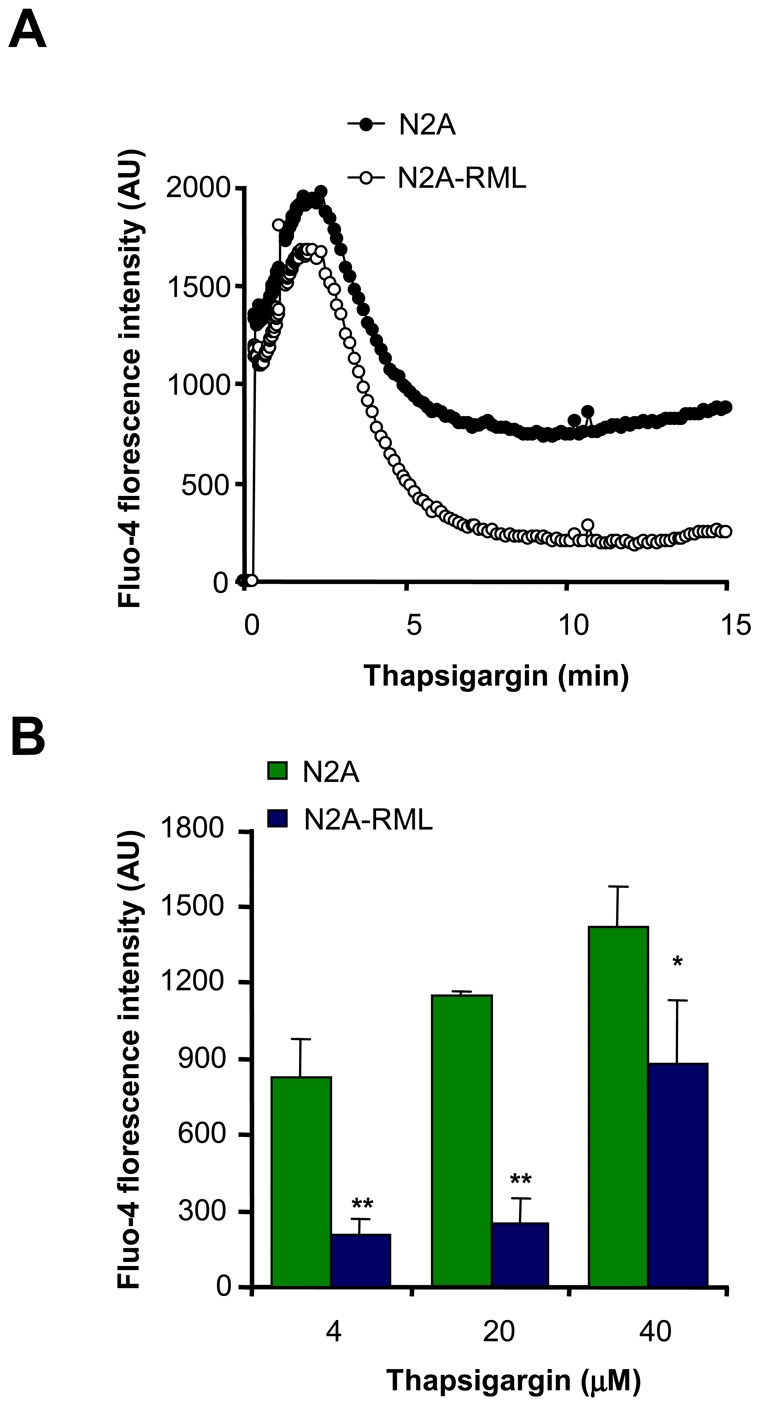
Altered calcium homeostasis in scrapie-infected cells. (**A**) N2a-RML and non-infected cells were loaded with the cytosolic calcium dye Fluo-4 and changes in fluorescence intensity were measured over time after addition of 4 µM thapsigargin using the FLIPR1 setup. Data represents the average of three independent experiments. (**B**) Calcium responses were analyzed after treatment with different concentrations of thapsigargin. Fluorescence levels after 10 min of treatment are shown. Mean and standard deviation is presented. Student t-test was used to analyze statistical significance (** *p*<0.01, * *p*<0.05). All determinations in panels A and B were made in the absence of extracellular calcium.

Based on the known connection between ER stress and perturbation of calcium homeostasis, we next explored the relative ER calcium content in N2a-RML cells. We monitored the passive release of calcium from the ER by inhibiting the SERCA pump with thapsigargin by using the calcium dye Fluo-4 (see controls for passive release figure S1C). All experiments were performed in the absence of extracellular calcium to specifically assess the contribution of intracellular calcium stores to cytosolic calcium signals. As shown in Figure 2A, N2a-RML cells presented diminished ER calcium release after thapsigargin treatment, which was dose-dependent (Figure 2B). As control, cells were pre-treated with thapsigargin for 30 min and then stimulated with A23187 in the absence of extracellular calcium, observed a dramatic attenuation of the release of calcium from the ER by thapsigargin tretament (not shown). These results suggest that prion replication affects ER calcium homeostasis. Based on these results, we then analyzed the subcelular distribution of PrP in N2a control and N2a-RML cells by subcellular fractionation using sucrose gradients. As previously described [21] an increased accumulation of PrP was observed at ER fractions (PDI positive) when cells were chronically infected with RML scrapie prions (Figure S2).

### SERCA overexpression sensitizes cells to acute exposure to infectious PrP^RES^

**Figure 3:**
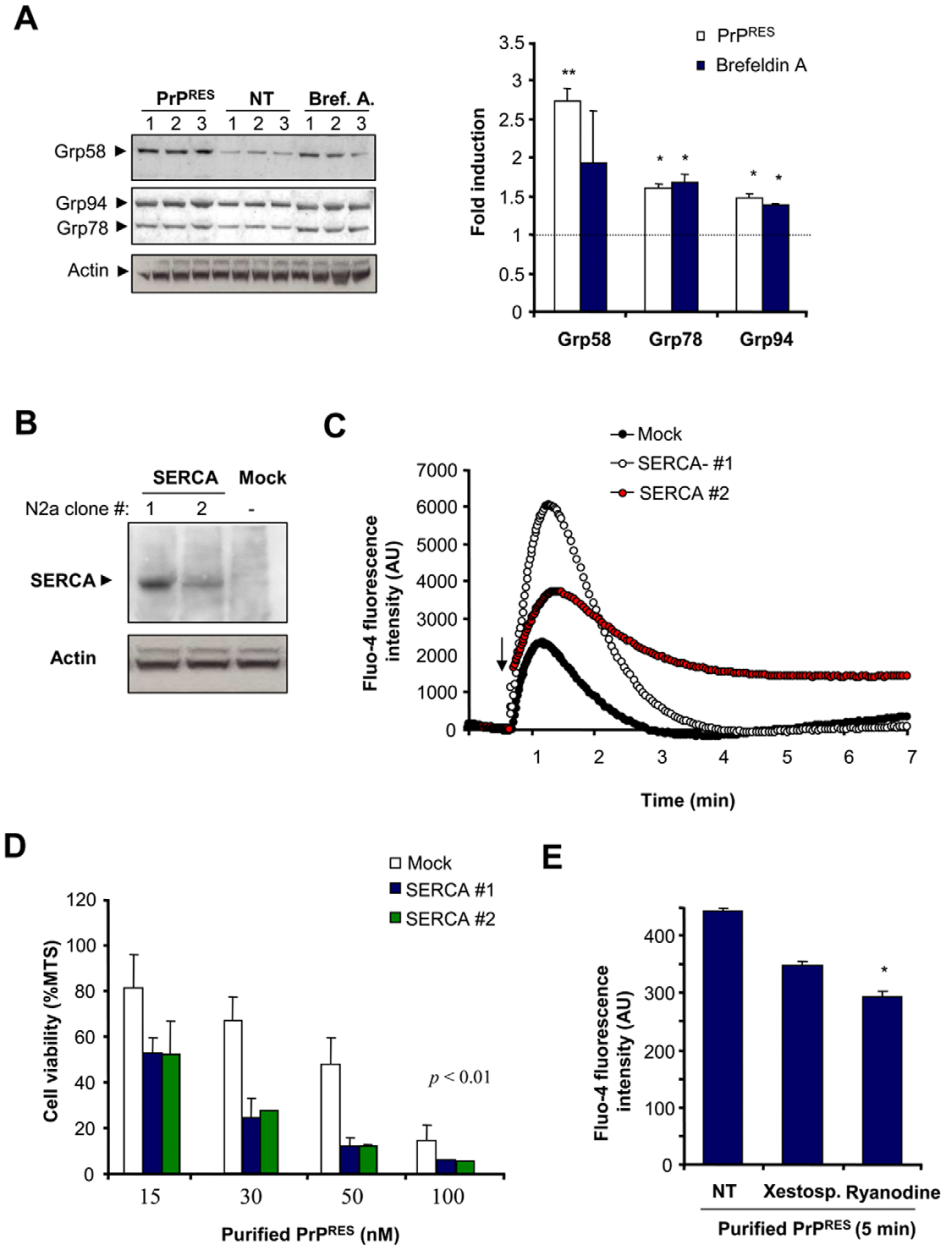
Role of ER calcium release after acute exposure to purified PrP^RES^ from scrapie-infected brains. (**A**) Neuro2a cells were treated for 27 h with brain derived PrP^RES^ (50 nM) or brefeldin A (12 µM), and the levels of Grp58, Grp78, and Grp94 were determined by Western blot. Three independent experiments are presented. Actin levels were monitored as loading control. Right panel: The protein band intensities were quantified and normalized with the expression of actin and the fold induction is presented in comparison with the average signal of non-treated cells. Values correspond to the mean and standard deviation. Student t-test was used to analyze statistical significance with control non-treated cells (** *p*<0.01, * *p*<0.05) (**B**) Neuro2a cells were stably transfected with an expression vector for SERCA, and its expression levels were determined by Western blot analysis. Two different cell clones and a control line transfected with empty pcDNA3.1 vector (Mock) are presented. (**C**) As control, the cell lines described in (A) were loaded with Fluo-4, and the release of ER calcium was monitored over time after addition of 300 nM A23187 (arrow) in the absence of extracellular calcium. Arbitrary units of fluorescence are shown (AU). (**D**) Cell lines expressing different amounts of SERCA pump and the control cell line (Mock) were treated with indicated concentrations of purified PrP^RES^ from 139A-scrapie infected brains. After 48 h of incubation, cell viability was analyzed with the MTS assay. Data represent mean and standard deviation of three experiments. *p* values were calculated with parametric t-test (**E**) Neuro2a cells were loaded with Fluo-4 and then pre-incubated with 10 µM ryanodine or 10 µM xestospongin C for 2 hours or left untreated. Calcium fluorescence was measured after 5 min of the addition of 200 nM of purified PrP^RES^. All determinations were performed in the absence of extracellular calcium. Data represent mean and standard deviation of three determinations. Student t-test was used to analyze statistical significance with control non-treated cells (* *p*<0.05).

We then addressed the possible role of ER stress and calcium homeostasis disturbance on an acute model of infectious PrDs. In this model cells were exposed to highly purified preparations of PrP^RES^ derived from brains of 139A scrapie-infected mice at the symptomatic stage (Figure S3A–C). Treatment of cells with brain-derived PrP^RES^ led to a significant induction of Grp58, Grp78 and Grp94 (Figure 3A), indicating the occurrence of ER stress. As positive control, cells were treated with brefeldin A. Using this system, incorporation of PrP to the cells was observed over time as monitored by Western blot in total protein extracts (Figure S3D). To further define a possible role of calcium in prion neurotoxicity we modulated ER calcium content by overexpressing SERCA, as previously described [53], [54]. We generated cell lines stably expressing SERCA and selected two lines for the analysis (Figure 3B). The levels of SERCA expression correlated well with the amount of calcium released from the ER after treatment with A23187 (Figure 3C), arachidonic acid or thapsigargin (not shown), indicating that this strategy has a functional effect on ER calcium metabolism in this cellular model. We then addressed the susceptibility of SERCA overexpressing cells. Using this acute model, purified PrP^RES^ led to significant cell death after 48 h of treatment in a dose-dependent manner using concentrations in the nanomolar range (Figure 3D). SERCA overexpressing cells were highly susceptible to PrP^RES^-induced cell death compared to control cells (Figure 3D).

The ER contains mainly two types of calcium channels, the inositol 1,4,5-triphosphate receptors (IP_3_Rs) and ryanodine receptors (RyRs) [55], which regulate the release of calcium into the cytoplasm. To study the possible contribution of these channels to PrP^RES^-induced calcium release, we pre-treated Neuro2a cells with xestospongin C or ryanodine, two known IP_3_Rs and RyRs inhibitors, respectively. Treatment of cells with these inhibitors decreased the cytosolic calcium increase after treatment of cells with brain-derived PrP^RES^ (Figure 3E). Unfortunately, it was not possible to study the effects of ER channel inhibitors on PrP^RES^ cytotoxicity because both compounds were highly toxic to Neuro2A cells after prolonged incubation (>24 h, data not show). This acute treatment may represent an additional contribution of cytosolic calcium to PrP^RES^ neurotocxicity due to mitochondrial calcium overload as suggested in models where cells were treated with micro molar concentrations of PrP peptides [40]. However, this acute experimental setting offers a useful measure of the impact of PrP^RES^ to ER calcium release by monitoring short term toxicity. Taken together, these results suggest that infectious PrP^RES^ alters ER calcium homeostasis.

### Expression of PrP mutants linked to CJD and FFI increase the susceptibility of cells to ER stress

**Figure 4:**
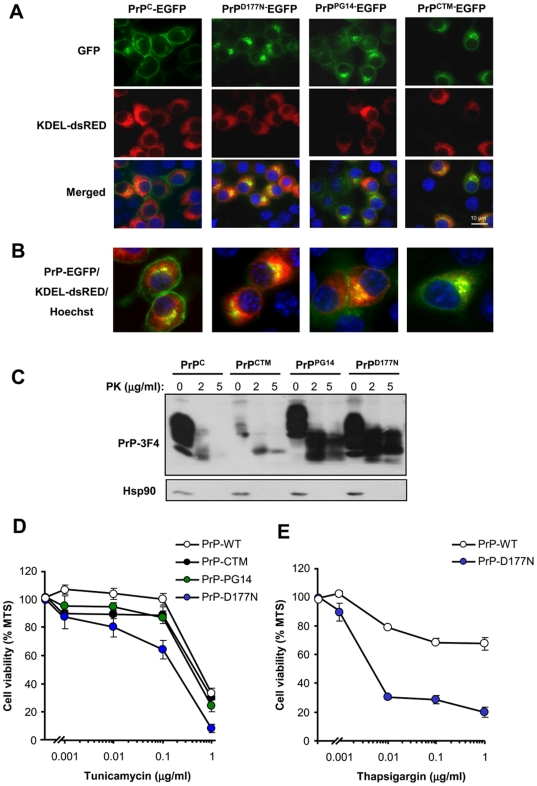
PrP mutants linked to familial PrDs are retained at the ER and increase the susceptibility of cells to ER stress. (**A**) EGFP fusion with PrP^C^ or the PrP mutants PrP^CTM^, PrP^D177^, and PrP^PG14^ were transiently expressed in Neuro2a cells. After 48 h, the subcellular localization of PrP (green) was visualized using confocal microscopy and co-localized with the ER-marker KDEL-dsRED (red). In addition the nucleus was stained with Hoechst (blue). (**B**) A higher magnification of cells analyzed under the same conditions described in panel A is presented. (**C**) To monitor PrP^RES^ levels, Neuro2a cells were transiently transfected with expression vectors for 3F4-tagged versions of PrP^C^ and mutant PrP. After 48 h, cell extracts were treated with indicated concentrations of proteinase K (PK) analyzed by Western blot. Hsp90 levels were monitored as loading control. (**D**) Neuro2a cells were stably transfected with expression vectors for 3F4-tag versions of PrP^C^, PrP^CTM^, PrP^D177^, and PrP^PG14^. Cells were grown in cell culture media containing 2% serum for 16 h and then exposed to different concentrations of tunicamycin. After 24 h, cell viability was determined with the MTS assay. Data represent mean and standard deviation of three determinations that are representative of three independent experiments. (**E**) In parallel, PrP^C^ and PrP^D177^ expressing cells were treated with indicated concentrations of thapsigargin and analyzed as described in D.

To investigate the role of ER stress in familial forms of PrD, we expressed two PrP mutant forms in Neuro2a cells, PrP^PG14^ and PrP^D177N/Met128^, which are linked to familial CJD and FFI respectively [2], [56]. In addition, we employed the neurotoxic mutant PrP^CTM^. We transiently transfected expression vectors for these PrP mutants and PrP^C^ as a control using EGFP fusion proteins, and then visualized their subcellular distribution by confocal microscopy and resistance to PK. As predicted, PrP^C^ was mainly located at the plasma membrane (Figure 4A and B). We also confirmed the partial retention of PrP^D177N^ and PrP^PG14^ at the ER after co-expression of PrP with the ER marker KDEL-dsRED (Figure 4A and B). Similarly, PrP^CTM^ predominantly accumulated at the ER (Figure 4A) and Golgi in our experimental system (co-stained with anti-GM130 antibodies, not shown). To monitor the possible generation of PrP^RES^, we transiently expressed 3F4-epitope tagged mutants in 293T cells. The addition of a 3F4 tag allowed us specifically detecting overexpressed PrP and not the endogenous protein. Total protein extracts were treated with two concentrations of PK and analyzed by Western blot. As shown in Figure 4C, expression of the PrP mutants lead to significant accumulation of PK-resistant PrP species. PrP^D177N^ displayed higher expression and increased PK-resistance (Figure 4C). Changes in the electrophoresis pattern of the mutants were observed as previously described [13], [16], corresponding to changes in the glycosylation pattern for PrP^CTM^ and PrP^D177N^, or a higher molecular weight for PrP^PG14^ due to the insertional mutation. After characterizing our cellular model, we generated Neuro2a cell lines stably expressing PrP^C^ or the three PrD-related mutants. Exposure of these cell lines to tunicamycin revealed that the expression of PrP^D177N^ increased the susceptibility to ER stress-induced cell death (Figure 4D). Expression of PrP^CTM^ or PrP^PG14^ expressing cells showed an intermediate phenotype, slightly enhancing their susceptibility to lower concentrations of tunicamycin treatment (Figure 4D). Treating PrP^D177N^ expressing cells with thapsigargin also confirmed an enhanced susceptibility to ER stress (Figure 4E). The changes in cell viability were small for PrP^CTM^ or PrP^PG14^ possibly because the stable selection of these neurotoxic mutants may lead to the elimination of a subpopulation of cells that are more susceptible to the pathological effects of the PrP mutants. In fact, the transient transfection of PrP mutants lead to ∼40% of cell death after 72 h of transfection as monitored by the MTS assay (not shown). Moreover, when we analyzed the levels of the different PrP constructs over time, we observed a decreased expression of PrP^CTM^ or PrP^PG14^ after stable selection, whereas higher PrP^D177N^ expression levels were maintained over time (Figure S4). Thus, these results suggest that PrP mutants related to familial PrDs, in addition to PrP^CTM^, alters the homeostasis of the ER to different extents, increasing the susceptibility to ER stress.

### Expression of mutant PrP forms alters ER calcium homeostasis

**Figure 5:**
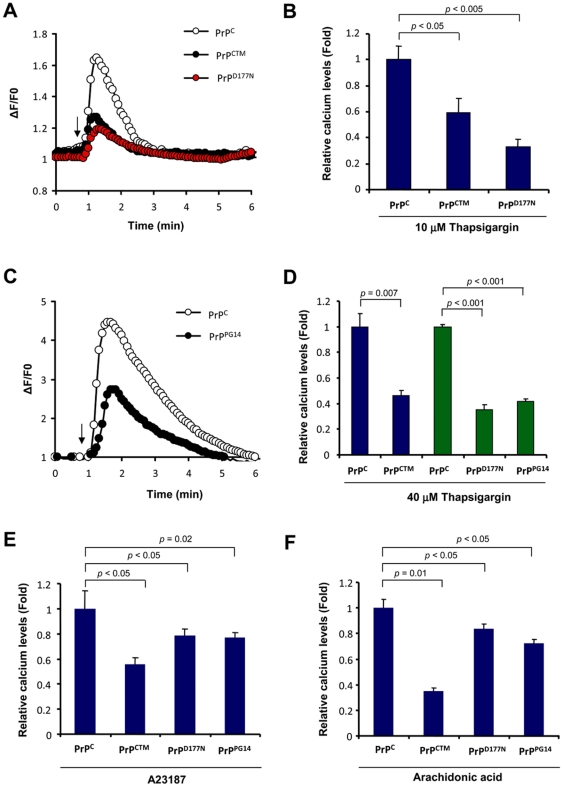
Expression of PrP mutants linked to familial PrDs alters ER calcium homeostasis. (**A**) Neuro2a cells stably transfected with PrP^C^, PrP^D177N^, or PrP^CTM^ were loaded with Fluo-4 and cytosolic calcium signals were monitored in cells exposed to 10 µM thapsigargin (arrow). All determinations were performed in the absence of extracellular calcium. A representative experiment is presented. (**B**) The maximum calcium signal from the experiment presented in panel A was quantified in a total of three independent experiments and normalized with the values obtained in PrP^C^ expressing cells (control). Mean and standard error is shown. (**C**) Neuro2a cells were stably transfected with PrP^C^ or PrP^PG14^ expression vectors and calcium signals were monitored as described in panel A after exposure to 40 µM thapsigargin (arrow). (**D**) The maximum calcium signal from experiment presented in panel A was quantified in a total of three independent experiments and normalized with the values obtained in PrP^C^ expressing cells (control). Mean and standard error is shown. Similar experiments were performed in cells expressing PrP^D177N^, or PrP^CTM^. (**E**) In parallel, cells expressing PrP^C^, PrP^CTM^, PrP^D177N^, or PrP^PG14^ were exposed to 10 µM A23187., or (**F**) 15 mM arachidonic acid and analyzed using conditions described in panel A. The maximum calcium signal from the experiment presented in panel A was quantified in a total of three independent experiments and normalized with the values obtained in PrP^C^ expressing cells (control). Mean and standard error is shown. In B, D, E and F, indicated *p* values were calculated with parametric t-test.

To address the possible impact of familial PrD mutant PrP on ER calcium homeostasis, we measured the relative ER calcium content in cells stably expressing three PrP mutants or wild type PrP^C^ after thapsigargin treatment in the absence of extracellular calcium. Treatment of Neuro2a cells expressing PrP^CTM^ or PrP^D177N^ with 10 µM thapsigargin led to a decreased release of ER calcium toward the cytosol compared to control PrP^C^ expressing cells (Figure 5A and B). Similar results were observed when cells were exposed to higher thapsigargin concentrations (40 µM; Figure 5C and D). These results were also recapitulated in cells expressing the PrP^PG14^ mutant (Figure 5C and D).

To further confirm that cells expressing mutant PrPs have alterations in ER homeostasis, we studied the effect of other ER calcium agonists. Treatment of PrP^CTM^, PrP^D177N^, or PrP^PG14^ expressing cells with A23187 in absence of extracellular calcium showed a substantial decrease on ER calcium release as compared with PrP^C^ expressing cells (Figure 5E). Similarly, treatment of all mutant expressing cells with arachidonic acid (Figure 1F), which induces IP_3_R-mediated calcium release, led to a lower cytosolic calcium increase. Together these results suggest that accumulation of PrP^RES^ at the ER in familial PrD models results in decreased ER calcium content.

## Discussion

The protein folding capacity of the ER is directly affected by different environmental factors including the redox status of the cell, ER calcium content and ATP levels. ER stress responses are observed in PrD models, and in human post mortem brain samples from patients affected with sporadic and new variant CJD [30], [31], [32], [33], [34], [35], [36], [39]. Remarkably, chronic ER stress is observed in many pathological conditions affecting the nervous system where the disease is etiologically linked to protein misfolding and abnormal protein aggregation [1].

The cellular events involved in perturbations of ER homeostasis in PrDs are not well understood. The main issue is rooted in the fact that prion replication mostly occurs at the plasma membrane (in lipids rafts) and during its trafficking and recycling through the endosomal pathway [5]. In addition, the majority of misfolded PrP in infectious PrD models accumulates in the brain extracellular space. Interestingly, different groups have shown that PrP^RES^ is also observed in intracellular compartments, where it could be poly-ubiquitinated and accumulated into cytosolic aggresomes and autophagosomes [25], [26]. In addition, there is evidence that PrP trafficking from the Golgi to the ER also contributes to the generation of PrP^RES^ in scrapie-infected cells [21]. We have reported the co-inmunoprecipitation of PrP^RES^ with ER-resident chaperones in scrapie-infected cells [34], suggesting that a subpopulation of infectious PrP^RES^ may actually target the ER lumen. Moreover, several studies have described the accumulation of mutant PrP molecules associated with hereditary PrDs at the ER and Golgi [7], [10], [11], [14], [15], [16], suggesting that some prion mutants may exert their pathological effects by affecting these organelles. Alternatively, different observations suggest that extracellular PrP^RES^ may lead to activation of intracellular signaling pathways that could indirectly impact the ER homeostasis and contribute to neuronal dysfunction [12]. However, the actual mechanism mediating PrP neurotoxicity in PrDs is not well understood.

In the present study we investigated the possible contribution of ER perturbations to prion pathogenesis in distinct PrD models resembling infectious and familial forms of the disease. Our data indicates that PrP misfolding leads to a higher susceptibility to ER stress and abnormal ER calcium homeostasis in chronically infected scrapie cells and in cells expressing PrP mutant forms associated with FFI and familial CJD. Similar results were observed when the neurotoxic mutant PrP^CTM^ was expressed in Neuro2a cells. In addition, we studied the impact of PrP^RES^ on calcium homeostasis using an acute PrD infectious model where exogenous PrP^RES^ was added to the cells at nanomolar concentrations. Using this system, we functionally addressed the impact of ER calcium by monitoring cell toxicity after expression of the ER calcium pump SERCA. In this acute setting, it is predicted that drastic and fast changes in cytosolic calcium will lead to mitochondrial calcium overload and apoptosis. In a chronic conditions, this release of calcium may occur slowly (i.e. in scrapie infected cells or neurons expressing familial PrP mutants), generating in the long term a decrease in the ER steady state calcium levels and ER stress. It may be feasibly that both cytosolic increased of calcium together with decreased steady state ER calcium content may synergise in the toxicity of misfolded PrP.

We speculate that a progressive and sustained release of ER calcium in neurons expressing misfolded PrP species may affect the protein folding status in this organelle, leading to basal ER stress, resulting in organelle failure and neuronal dysfunction. At the same time, increased levels of calcium in the cytoplasm may perturb several signaling pathways implicated in controlling neuronal function and survival. Indeed, our recent results suggest that PrP^RES^ formation leads to an hyperactivation of the calcium-dependent phosphatase calcineurin *in vivo*, leading to dephosphorylation of CREB and BAD [58]. Strikingly, treatment of prion infected mice at the clinical phase of the disease with the FDA-approved calcineurin inhibitor FK506 reduced neurodegeneration, leading to improvement on behavioral alterations and increase animal survival [58], suggesting a functional role of calcium in synaptic dysfunction and neuronal death.

The ER is a primary compartment for PrP^C^ folding and also plays a crucial role in the generation of pathogenic PrP protein conformations in familial PrDs. Therefore, it is essential to understand the folding pathways of PrP^C^ because this information may give clues about the mechanism underlying sporadic forms of CJD (the most common PrD in humans) where alteration in the folding/quality control process or the ER environment may be a key event in initiating the pathology. In support of this hypothesis, post-mortem studies of sporadic and new variant CJD brain samples demonstrated upregulation of ER stress-inducible foldases [5], [30], [31]. Several studies indicate that alterations of the ER homeostasis or inhibition of the proteasome, which blocks ERAD of misfolded PrP^C^, lead to accumulation of abnormally folded PrP^C^ that is partially resistant to proteases and insoluble in non-denaturing detergents [6], [22], [27], [37], [57]. These findings together with the results described in the current study suggest that ER factors and/or the ER environment, including ER calcium concentrations, are key determinants of PrP misfolding and the generation of neurotoxic species. The early signaling events mediating ER calcium abnormalities by the accumulation of PrP^RES^ remain to be determined. Interactions between misfolded PrP and the proteins modulating calcium transport (i.e. SERCA, IP3Rs and/or RyRs) may explain part of these phenotypes as suggested in Huntington's disease models [59], [60]. The findings presented here may also contribute to a better understanding of the pathogenesis of other diseases affecting the nervous system related to protein misfolding and ER stress.

## Materials and Methods

### Materials

Tunicamycin, A23187, thapsigargin, calphostine, xestospongin C, and ryanodine were purchased from Calbiochem EMB Bioscience Inc. (Darmstadt, Germany). Cell medium, fetal calf serum and antibiotics were obtained from Life Technologies (Maryland, USA). Arachidonic acid and dantrolene were obtained from Sigma (Basel, Switzerland). Fluo-4 was purchased from Molecular Probes. Superfect and plasmid purification kits were pursed from Qiagene (HiSpeed Plasmid Midi Kit).

### Cell culture and viability assays

Neuro2A cells were obtained from ATCC and were cultured in DMEM supplemented with 10% fetal calf serum and antibiotics (10′000 U/ml Penicillin, 10 µg/ml streptomycin), at 37°C and 5% CO_2._ For cell viability analysis, cells were grown in collagen IV coated 96-well plates for 24 h in cell culture medium in 1% serum before addition of the agonist. Cell viability was quantified using 3-(4, 5-dimethylthazol-2-yl)-5-3-carboxymethoxy-phenyl)-2-(4-sulfophenyl)-2H-tetrazolium (MTS) according to the recommendations of the supplier (Promega, CellTiter96® Aqueous, Madison, WI). In addition, cellular death by apoptosis was quantified by nuclear staining with Hoechst33342.

### PrP^RES^ purification from the brain of scrapie infected mice

PrP^RES^ was purified from mice infected with 139A scrapie as previously described [61]. Experimental animal protocols for animal use has been reviewed and approved by the Institutional Review Board's Animal Care and Use Committee of the Faculty of Medicine of the University of Chile (approved protocol CBA # 0232 FMUCH). Brain tissue (approximately 12 brains per preparation) was homogenized with a manual potter of 20 ml in PBS (final concentration 50% weight/volume) containing a protease inhibitor cocktail. After homogenization, an equivalent volume of a solution containing 20% salkosyl and 0.05% octanol was added. The brain extract was incubated for 15 min at room temperature with constant agitation in a wheel rotor. After this step, non-disrupted tissue was eliminated by centrifugation at 7.000× g for 15 min. The supernatant was collected and 1/3 volume of 0.1% SB3-14 was added to the brain homogenate, mixed and centrifuged at 50.000 r.p.m. in a Ti60 rotor (Beckman) for 2 h at 4°C. After centrifugation, the pellets were collected and resuspended by sonication in 10% NaCl, 0.1% SB3-14. The homogenized pellet was loaded over a sucrose solution (20% sucrose, 0.1% SB-314) and centrifuged at 80.000 r.p.m. for 2 h at 4°C in a TL100 rotor (Beckman). The pellet was collected, washed in PBS and resuspended by sonication in PBS containing 0.1% SB-314. Thereafter, samples were treated with PK (50 µg/ml) for 2 h followed by another sucrose step separation after centrifugation at 80.000 r.p.m. for 2 h. The pellet was washed four times with sterile PBS and resuspended in 400 µl of PBS by sonication. After this step, purity was estimated to be higher than 90% as estimated by silver staining and mass spectrometric analysis. PrP^RES^ concentration was estimated by western blot analysis, comparing in the same blot the signal intensity of different dilutions of the purified protein with known concentrations of the recombinant mouse PrP^C^, purchased from Prionics Inc (Zurich, Switzerland).

### Plasmids and cell transfections

Expression vector containing SERCA from rabbit was kindly provided by Frederica Del Monte (University of Toronto, Canada). Expression plasmid containing catalytically inactive caspase-12 mutant lacking its N-terminal pro-domain (amino acids 1-94, pC12DN) was provided by R. Rao (California). Expression vectors of 3F4 tagged PrP mutants (PrP^CTM^, PrP^PG14^, and PrP^D177N^) and GFP fusion proteins were provided by David Harris (Washington University). The generation of PrP^C^-3F4 and PrP^C^-EGFP constructs was previously described [57]. Co-localization of PrP with ER was assessed by co-transfection with pDsRed-ER (Clontech Laboratories, cat. 6982-1). Stably-expressing Neuro2a cells were produced by transfection using SuperFect kit (Qiagene, Valencia, CA) following the manufacturer's instructions. After 48 h of transfection, cells were selected using hygromicin (1.5 mg/ml) or G418 (1.3 mg/ml). Individual clones of Neuro2a cells expressing different levels of SERCA, were obtained by limiting dilution as described before [30].

### Generation of Neuro2A cells chronically infected with RML scrapie prions

Neuro2a cells can be infected with PrP^RES^ although the response and the stability of the infected cells has proved variable, suggesting heterogeneity may exist in the original cell line [62], [63]. In order to establish a stable chronically infected cell, we separated the original culture in subclones by limiting dilution. A growing culture was diluted to a density of 5 cells/ml and 100 µl was transferred to individual wells of a 96 well plate and cultured for 1 week. The individual cultures were examined microscopically to determine those wells which contained a single focus of growing cells. The single cell derived cultures were then transferred to 24 well plates and serially passaged every 3–4 days at 1∶15 dilution to maintain stocks. Single clone cultures were tested for sensitivity to infection by the RML strain of PrP^RES^. To do this, 4 µl of a 10% late stage infected brain extract was added per well of newly passaged cells, and the cultures were left for a further 4 days to reach confluence. Cells were serially passaged thereafter in the absence of PrP^RES^. Tests showed that all trace of the initial inoculum disappeared by passage 4. At this and later passages individual cultures were tested for the presence of PrP^RES^ by dot blotting as previously described [62].

### Measurement of intracellular calcium

For biosecurity reasons, in experiment with RML infected cells or cells treated with purified PrP^RES^, the changes in intracellular calcium levels were measured using the automatized FLIPR1 machine (Molecular devices, Sunnyvale, CA) by the use of the fluorescent dye Fluo-4, which shows increased fluorescence at 515–535 nm after calcium binding. 10^5^ cells per well were grown on 96-black wells plate coated with collagen IV for 24 h and serum was decreased to 1% for a further 24 h. Cells were loaded with Fluo-4 at a final concentration of 10 µg/mL. After 120 min of incubation at 37°C, the loaded cells were washed twice with FLIPR buffer (150 mM NaCl, 5 mM KCl, 1 mM MgCl_2_, 10 mM Hepes, 10 mM glucose) in the absence of extracellular calcium and plates were mounted on the FLIPR1 setup. Fluorescence emission was quantified every 5 seconds for a total time of 30 min. The basal fluorescence of the dye was usually determined before the addition of the samples and was assigned a value of zero. The agonists and inhibitor were added automatically by the FLIPR1 setup, and were prepared in an independent 96-well plate. To determine the origin of intracellular calcium, cells were pre-treated with thapsigargin (5 µM), a ryanodine mix (10 µM) or xestospongin C for 15 min before addition of the agonists.

In experiment with PrP mutants, cells were grown in coverslips and loaded with Fluo-4 AM (3 µM) for 30 min at room temperature. Coverslips were mounted in a 1 ml capacity chamber and washed three times with calcium-free buffer (150 mM NaCl, 5 mM KCl, 1 mM MgCl_2_, 10 mM Hepes, 10 mM glucose and 5 mM EGTA, pH 7.4). Calcium signals were recorded using an IX-81 inverted microscope for fluorescence measurements (DSU, Olympus), equipped with a 150-W xenon lamp (Olympus MT-20). Fluo-4 fluorescence was excited and detected with a FITC filter cube, using a 40x/1.4 NA oil immersion objective. Changes in [Ca^2+^]_i_ were measured in a field-of-view consisting of 15–30 cells. Images were acquired every 5 seconds and analyzed using CellR (Olympus) and NIH ImageJ software. The mean intensities of small cellular areas of interest were collected as F(t) and the background intensity was subtracted, using a same-size region of interest outside the cell, yielding F(t)s. The final signal was normalized to baseline fluorescence F(0), as [F(t)s − F(0)]/F(0) [64], [65].

### SDS-PAGE and Western Blot Analysis

Cells were homogenized on ice in RIPA buffer (20 mM Tris pH 8.0, 150 mM NaCl, 0.1% SDS, 0.5% DOC, 0.5% triton X-100) containing a protease inhibitor cocktail (Roche, Basel, Germany) as previously described [66]. Protein concentration was determined by micro-BCA assay (Pierce, Rockford, IL). The equivalent of 30–50 µg of total protein was generally loaded onto 10% SDS-PAGE minigels (Novex NuPage, Invitrogen Life Technologies, Basel, Switzerland) and analysed by Western blotting as described. The following antibodies and dilutions were used: 6H4 anti-PrP, 1∶10,000 (Prionics, Zurich, Switzerland), anti-Caspase-12, 1∶2,000 (Exalpha, Watertown, USA); anti-GRP78/Bip, anti-Grp58/ERp57 and anti-Grp94 1∶2,000 (StressGene, San Diego, CA); anti-actin, 1∶2,000 and Hsp90, 1∶3000 (Santa Cruz), anti-3F4 antibody 1∶5000 (Abcam). After incubation with the primary antibody, membranes were incubated for 1 h at room temperature with horseradish peroxidase-coupled second antibodies diluted 1∶10,000 in washing buffer. After washing, specifically bound antibodies were detected by enhanced chemiluminescence assay (Amersham Biosciences, Cardiff, UK).

### Subcellular Fractionation

To separate and enrich ER membranes, Neuro2a cells were homogenized by using a stainless steel ball-bearing homogenizer in 0.25 M sucrose, 10 mM Tris-HCl, pH 7.4, 1 mM magnesium acetate, and a protease inhibitor mixture in a final concentration of 1 volume of cell pellet per 5 volumes of homogenizing medium. Sucrose gradients were performed as described in [21]. 1-ml fractions were collected from the top of each gradient, assayed for protein content, and methanol-precipitated. After centrifugation at 14,000 rpm for 20 min, the pellets were resuspended in SDS loading buffer.

### Statistical analysis

Data was analyzed by parametric t-test (two-tailed) and significance was expressed as follow: * *P*<0.05; ** *P*<0.01; *** *P*<0.005. For the analysis the program SigmaPlot and GraphPad were employed.

## Supporting Information

Figure S1 **Control experiments. (A) Replication of PrPRES at expenses ofendogenous PrPC is required to increase the susceptibility to ER stress.** Twodifferent Neuro2a clones were selected by their property to sustain replication of RMLprions (N2a-RML) or that are resistant to replication (N2a-RML-Ins). Cell were exposedto RML scrapie prion and after several weeks in culture, they were treated with 12 mMbrefeldin A (Bref. A) or 40 nM A23187. After 48h cell viability was monitored using theMTS assay. Mean and standard deviation is presented of three determinations. **(B) Expression of a caspase-12 dominant negative mutant form protect against ERstress.** Left panel: Neuro2 cells were stably transfected with empty pCDNA.3 vector oran expression vector for a caspase-12 dominant negative (C289A) construct. Then,cell viability was monitored after exposure of cells to 12 mM brefeldin A or 5 mMthapsigargin for 48h using the MTS assay. Data represent mean and standarddeviation of three determinations. Right panel: Expression levels of caspase-12 andactin are presented as controls. **(C) Thapsigargin treatment triggers passive relatedof ER calcium, not affected by inhibition of IP_3_R.** Neuro2a cells were loaded withFluo-4 and cytosolic calcium signals were monitored in cells exposed to 10 mMthapsigargin (arrow). Cells were pretreated or not with 1 mM Xestospongine B (IP3Rinhibitor) for 1h or 50 mM dantrolen (RYR inhibitor) for 30 min. All determinations wereperformed in the absence of extracellular calcium. A representative experiment ispresented.
(PDF)

Figure S2 **Increased accumulation PrP at ER fractions in Neuro2a cells infectedwith RML scrapie prions.** Postnuclear cell extracts from Neuro2a control and RMLinfectedcells were fractionated on a sucrose gradient to separate ER fractions asdescribed in material and methods. Total proteins present in fractions of 1 ml wereprecipitated and analyzed by Western blot. Total PrP levels were monitored in eachfraction. As control to identify ER-enriched fractions, the distribution of PDI wasassessed by Western blot.
(PDF)

Figure S3 **Purification of PrPRES from 139A-scrapie infected brains.** (**A**) Schematicrepresentation of the preparation steps used to purify PrPRES from 139A-scrapieinfected brains (described in material and methods). (**B**) Qualitative analysis of theenrichment on PrPRES during purification procedure. Equivalent samples from differentsteps of the purification process were analyzed by western blot or by silver staining ofthe total proteins presented in each sample. Samples were loaded in the followingorder: **1:** 10% brain homogenate in PBS. **2:** Sarcosyl solubilization. **3:** Sarcosylextraction, pellet. **4:** Pellet obtained after sucrose gradient before PK treatment. **5:** Pellet obtained after sucrose gradient after PK treatment. **6:** 500 ng of recombinantPrPC, used as a positive control. (**C**) Quantification of PrPRES concentration. Knownamounts of recombinant PrPC were compared with different dilutions of purified PrPRESby western blot analysis (left panel). The band intensity was quantified to estimate theconcentration of PrPRES by comparison to the values obtained with the calibration curveof recombinant PrPC (right panel). R2 corresponds to the linear regression coefficient. (**D**) Neuro2a cells were treated with 1 mg/ml brain derived PrPRES for indicated timepoints and then washed extensively with PBS. Then cells were collected bytripsinization and further washed in PBS by centrifugation. PrP levels were monitoredby Western blot in total protein extracts. The molecular weight of PrP corresponds tothe PK-resistant core, indicating the detection of the exogenously added brain-derivedPrP. In this assay, PrPRES oligomers are also observed (*). Actin levels were monitoredas control.
(PDF)

Figure S4 **Stable expression of prion mutants.** Neuro2A cells were transfected withindicated PrP expressing vectors containing the 3F4 tag epitope, and then selectedwith G418 (1.3 mg/ml). The expression levels for each PrP version was assessed overtime by Western blot analysis during early selection (∼1 week, upper panel) or afterstable selection (3 week, middle panel; 4 weeks, bottom panel). As loading control anon specific band is presented from the 3F4 Western blot. Cells presented in the rightpanel were used to the viability assays.
(PDF)
