# Correction: Prion Protein Misfolding Affects Calcium Homeostasis and Sensitizes Cells to Endoplasmic Reticulum Stress

**DOI:** 10.1371/annotation/4087ecad-630d-4436-a301-257b13441156

**Published:** 2011-03-21

**Authors:** Mauricio Torres, Karen Castillo, Ricardo Armisén, Andrés Stutzin, Claudio Soto, Claudio Hetz

There is an error in the funding section. The grant FONDECYT number 1070444 should be: 1100176.

