# Correction: Prion Protein Misfolding Affects Calcium Homeostasis and Sensitizes Cells to Endoplasmic Reticulum Stress

**DOI:** 10.1371/journal.pone.0262628

**Published:** 2022-01-11

**Authors:** Mauricio Torres, Karen Castillo, Ricardo Armisén, Andrés Stutzin, Claudio Soto, Claudio Hetz

An error was made in preparing the Actin panel of Fig 3A of this article [1]: lanes 1, 11, and 12 from the original blot should not have been included. An updated Fig 3 is provided here in which this has been corrected. Lanes 2–10 of the Actin blot were loaded with the same volumes of the same protein samples as those included in lanes 1–9 of other panels in the figure. Parallel blots with the same samples were probed with the indicated antibodies. The available quantitative data underlying graphs in Fig 3 are in S1 File of this notice. The original blots for Fig 3 and the data supporting some other results reported in the article [1] are no longer available.

The authors apologize for the Fig 3A error that was generated during figure preparation. The conclusions of the experiment are not altered by this error.

**Fig 3:**
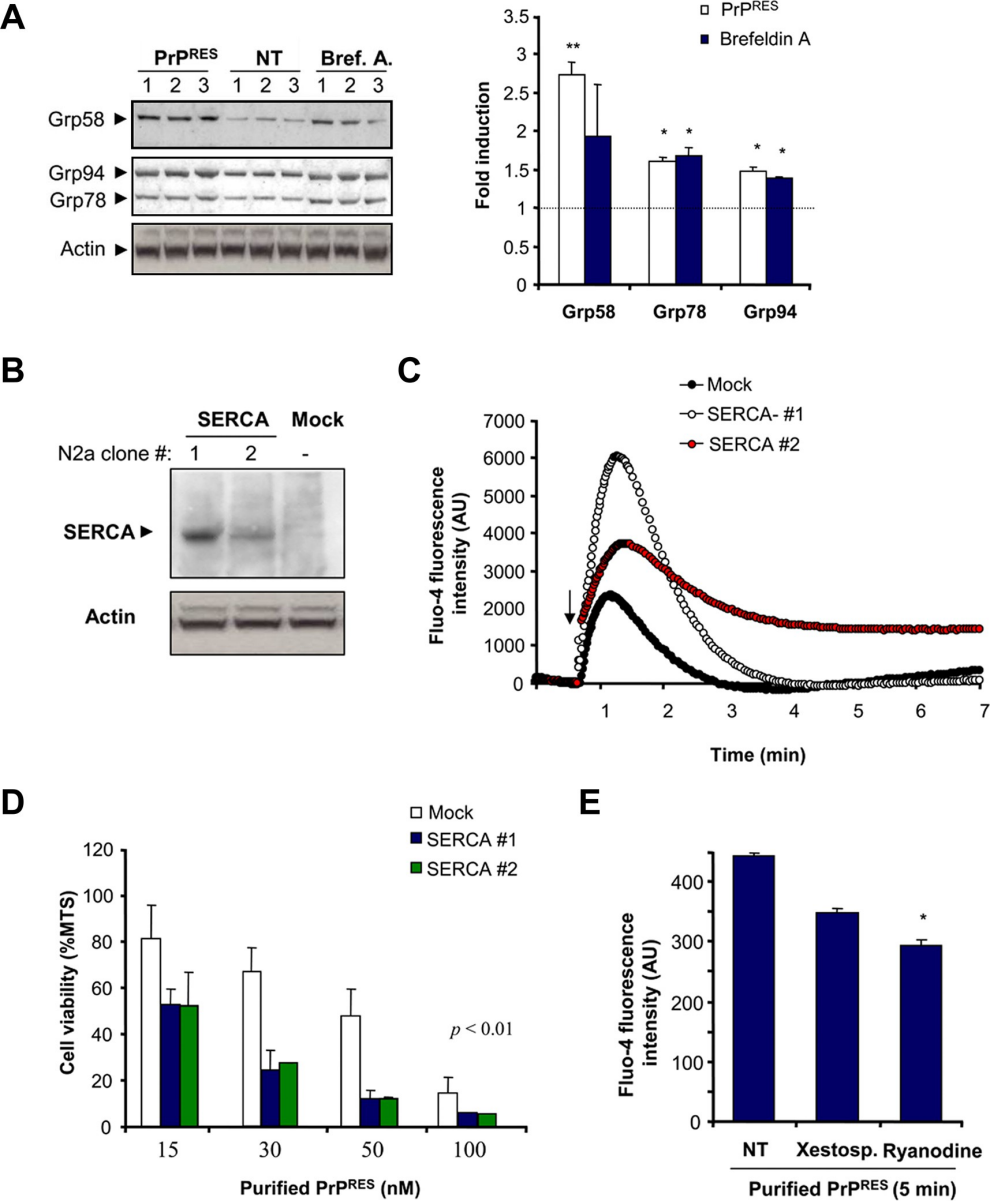
Role of ER calcium release after acute exposure to purified PrP^RES^ from scrapie-infected brains. (**A**) Neuro2a cells were treated for 27 h with brain derived PrP^RES^ (50 nM) or brefeldin A (12 µM), and the levels of Grp58, Grp78, and Grp94 were determined by Western blot. Three independent experiments are presented. Actin levels were monitored as loading control. Right panel: The protein band intensities were quantified and normalized with the expression of actin and the fold induction is presented in comparison with the average signal of non-treated cells. Values correspond to the mean and standard deviation. Student t-test was used to analyze statistical significance with control non-treated cells (** *p*<0.01, * *p*<0.05) (**B**) Neuro2a cells were stably transfected with an expression vector for SERCA, and its expression levels were determined by Western blot analysis. Two different cell clones and a control line transfected with empty pcDNA3.1 vector (Mock) are presented. (**C**) As control, the cell lines described in (A) were loaded with Fluo-4, and the release of ER calcium was monitored over time after addition of 300 nM A23187 (arrow) in the absence of extracellular calcium. Arbitrary units of fluorescence are shown (AU). (**D**) Cell lines expressing different amounts of SERCA pump and the control cell line (Mock) were treated with indicated concentrations of purified PrP^RES^ from 139A-scrapie infected brains. After 48 h of incubation, cell viability was analyzed with the MTS assay. Data represent mean and standard deviation of three experiments. *p* values were calculated with parametric t-test (**E**) Neuro2a cells were loaded with Fluo-4 and then pre-incubated with 10 µM ryanodine or 10 µM xestospongin C for 2 hours or left untreated. Calcium fluorescence was measured after 5 min of the addition of 200 nM of purified PrP^RES^. All determinations were performed in the absence of extracellular calcium. Data represent mean and standard deviation of three determinations. Student t-test was used to analyze statistical significance with control non-treated cells (* *p*<0.05).

## Supporting information

S1 File Quantitative data underlying Fig 3A, 3C, 3D, and 3E. Individual-level data for Fig 3D are no longer available. For Fig 3E, raw data are provided only for two replicates although the graph in Fig 3E reports data from three replicates.
(XLSX)
