# Supplementary material for: Prion Protein Misfolding Affects Calcium Homeostasis and Sensitizes Cells to Endoplasmic Reticulum Stress
Source: PLoS One. 2010 Dec 29;5(12):e15658. doi: 10.1371/journal.pone.0015658 (PMC3012133; doi:10.1371/journal.pone.0015658)
Supplement: Figure S1 — Control experiments. (A) Replication of PrPRES at expenses ofendogenous PrPC is required to increase the susceptibility to ER stress. Twodifferent Neuro2a clones were selected by their property to sustain replication of RMLprions (N2a-RML) or that are resistant to replication (N2a-RML-Ins). Cell were exposedto RML scrapie prion and after several weeks in culture, they were treated with 12 mMbrefeldin A (Bref. A) or 40 nM A23187. After 48h cell viability was monitored using theMTS assay. Mean and standard deviation is presented of three determinations. (B) Expression of a caspase-12 dominant negative mutant form protect against ERstress. Left panel: Neuro2 cells were stably transfected with empty pCDNA.3 vector oran expression vector for a caspase-12 dominant negative (C289A) construct. Then,cell viability was monitored after exposure of cells to 12 mM brefeldin A or 5 mMthapsigargin for 48h using the MTS assay. Data represent mean and standarddeviation of three determinations. Right panel: Expression levels of caspase-12 andactin are presented as controls. (C) Thapsigargin treatment triggers passive relatedof ER calcium, not affected by inhibition of IP3R. Neuro2a cells were loaded withFluo-4 and cytosolic calcium signals were monitored in cells exposed to 10 mMthapsigargin (arrow). Cells were pretreated or not with 1 mM Xestospongine B (IP3Rinhibitor) for 1h or 50 mM dantrolen (RYR inhibitor) for 30 min. All determinations wereperformed in the absence of extracellular calcium. A representative experiment ispresented. (PDF) [file pone.0015658.s001.pdf]

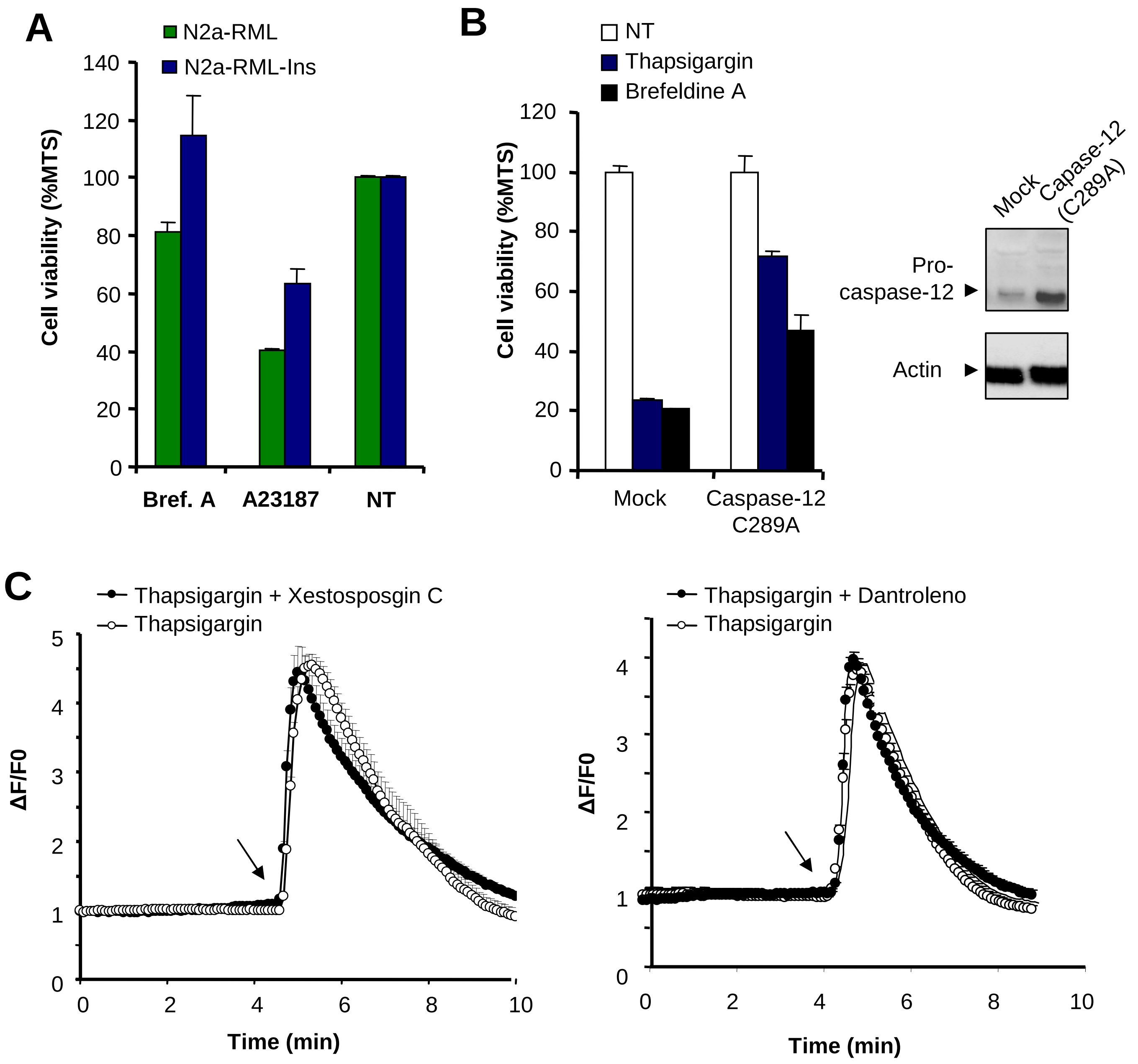

Figure S1. Control experiments. (A) ***Replication of PrP^{RES} at expenses of endogenous PrP^{C} is required to increase the susceptibility to ER stress.*** Two different Neuro2a clones were selected by their property to sustain replication of RML prions (N2a-RML) or that are resistant to replication (N2a-RML-Ins). Cell were exposed to RML scrapie prion and after several weeks in culture, they were treated with 12 µM brefeldin A (Bref. A) or 40 nM A23187. After 48h cell viability was monitored using the MTS assay. Mean and standard deviation is presented of three determinations. **(B)** ***Expression of a caspase-12 dominant negative mutant form protect against ER stress.*** Left panel: Neuro2 cells were stably transfected with empty pCDNA.3 vector or an expression vector for a caspase-12 dominant negative (C289A) construct. Then, cell viability was monitored after exposure of cells to 12 µM brefeldin A or 5 µM thapsigargin for 48h using the MTS assay. Data represent mean and standard deviation of three determinations. Right panel: Expression levels of caspase-12 and actin are presented as controls. **(C)** ***Thapsigargin treatment triggers passive related of ER calcium, not affected by inhibition of IP$_3$R.*** Neuro2a cells were loaded with Fluo-4 and cytosolic calcium signals were monitored in cells exposed to 10 µM thapsigargin (arrow). Cells were pretreated or not with 1 µM Xestospongine B (IP$_3$R inhibitor) for 1h or 50 µM dantrolen (RYR inhibitor) for 30 min. All determinations were performed in the absence of extracellular calcium. A representative experiment is presented.

Torres et al., Figure S1
