# Supplementary material for: Prion Protein Misfolding Affects Calcium Homeostasis and Sensitizes Cells to Endoplasmic Reticulum Stress
Source: PLoS One. 2010 Dec 29;5(12):e15658. doi: 10.1371/journal.pone.0015658 (PMC3012133; doi:10.1371/journal.pone.0015658)
Supplement: Figure S2 — Increased accumulation PrP at ER fractions in Neuro2a cells infectedwith RML scrapie prions. Postnuclear cell extracts from Neuro2a control and RMLinfectedcells were fractionated on a sucrose gradient to separate ER fractions asdescribed in material and methods. Total proteins present in fractions of 1 ml wereprecipitated and analyzed by Western blot. Total PrP levels were monitored in eachfraction. As control to identify ER-enriched fractions, the distribution of PDI wasassessed by Western blot. (PDF) [file pone.0015658.s002.pdf]

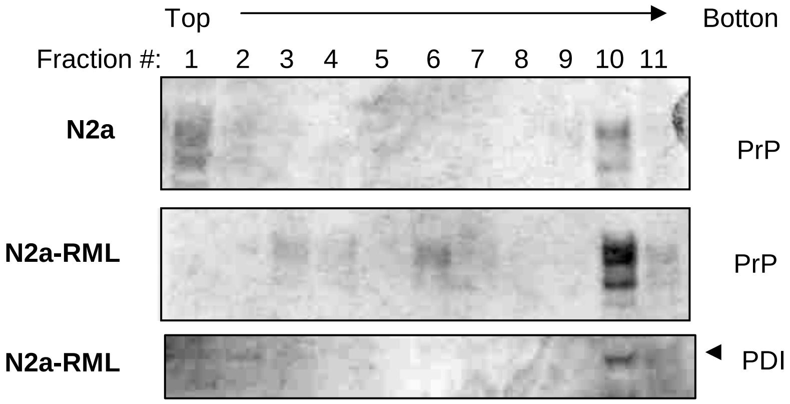

Figure S2. Increased accumulation PrP at ER fractions in Neuro2a cells infected with RML scrapie prions. Post-nuclear cell extracts from Neuro2a control and RML-infected cells were fractionated on a sucrose gradient to separate ER fractions as described in material and methods. Total proteins present in fractions of 1 ml were precipitated and analyzed by Western blot. Total PrP levels were monitored in each fraction. As control to identify ER-enriched fractions, the distribution of PDI was assessed by Western blot.

Torres et al., Figure S2
