# Supplementary material for: Prion Protein Misfolding Affects Calcium Homeostasis and Sensitizes Cells to Endoplasmic Reticulum Stress
Source: PLoS One. 2010 Dec 29;5(12):e15658. doi: 10.1371/journal.pone.0015658 (PMC3012133; doi:10.1371/journal.pone.0015658)
Supplement: Figure S3 — Purification of PrPRES from 139A-scrapie infected brains. (A) Schematicrepresentation of the preparation steps used to purify PrPRES from 139A-scrapieinfected brains (described in material and methods). (B) Qualitative analysis of theenrichment on PrPRES during purification procedure. Equivalent samples from differentsteps of the purification process were analyzed by western blot or by silver staining ofthe total proteins presented in each sample. Samples were loaded in the followingorder: 1: 10% brain homogenate in PBS. 2: Sarcosyl solubilization. 3: Sarcosylextraction, pellet. 4: Pellet obtained after sucrose gradient before PK treatment. 5: Pellet obtained after sucrose gradient after PK treatment. 6: 500 ng of recombinantPrPC, used as a positive control. (C) Quantification of PrPRES concentration. Knownamounts of recombinant PrPC were compared with different dilutions of purified PrPRESby western blot analysis (left panel). The band intensity was quantified to estimate theconcentration of PrPRES by comparison to the values obtained with the calibration curveof recombinant PrPC (right panel). R2 corresponds to the linear regression coefficient. (D) Neuro2a cells were treated with 1 mg/ml brain derived PrPRES for indicated timepoints and then washed extensively with PBS. Then cells were collected bytripsinization and further washed in PBS by centrifugation. PrP levels were monitoredby Western blot in total protein extracts. The molecular weight of PrP corresponds tothe PK-resistant core, indicating the detection of the exogenously added brain-derivedPrP. In this assay, PrPRES oligomers are also observed (*). Actin levels were monitoredas control. (PDF) [file pone.0015658.s003.pdf]

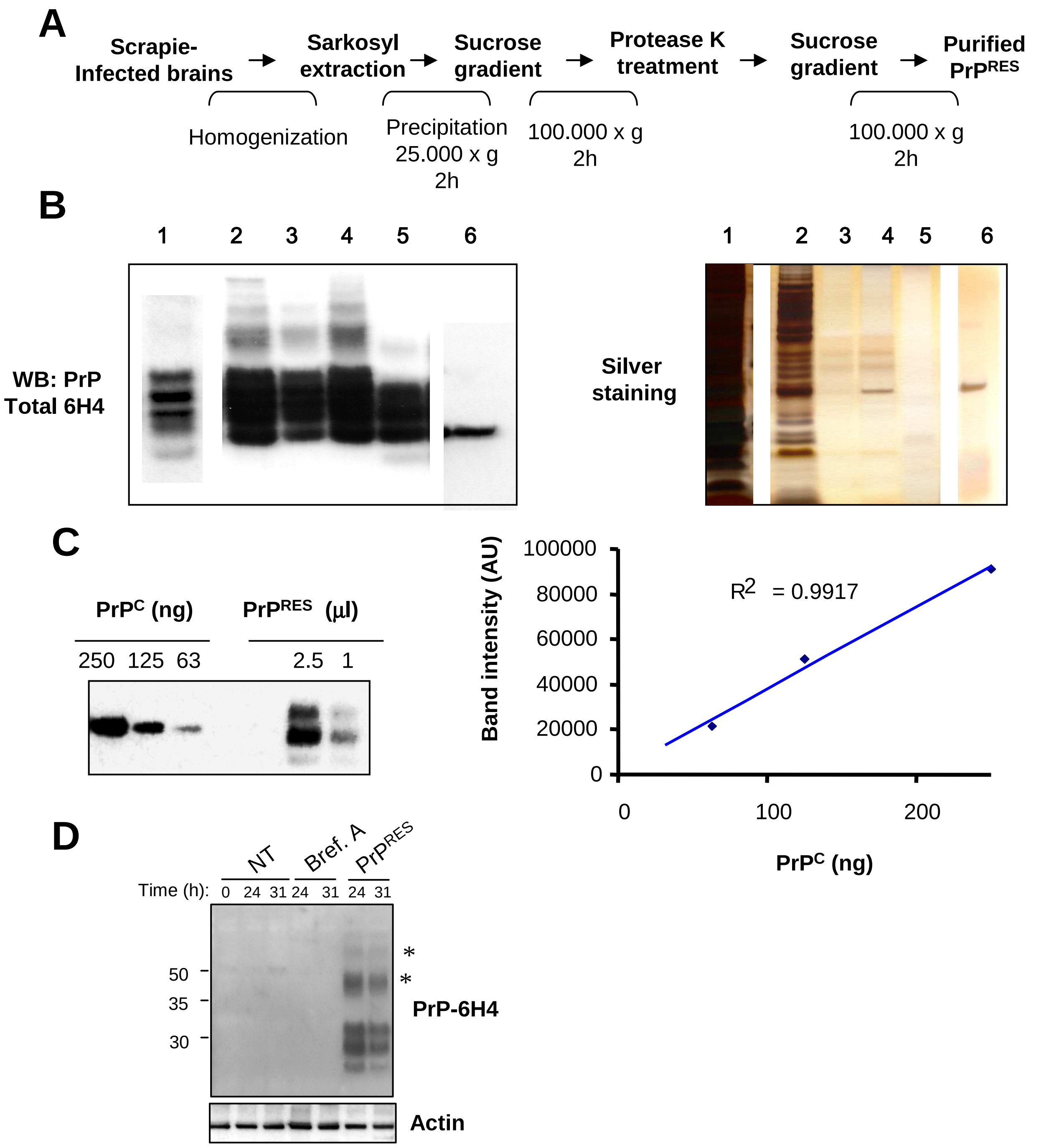

Torres et al., Figure S3

Figure S3. Purification of PrP^{RES} from 139A-scrapie infected brains. (A) Schematic representation of the preparation steps used to purify PrP^{RES} from 139A-scrapie infected brains (described in material and methods). **(B)** Qualitative analysis of the enrichment on PrP^{RES} during purification procedure. Equivalent samples from different steps of the purification process were analyzed by western blot or by silver staining of the total proteins presented in each sample. Samples were loaded in the following order: **1:** 10% brain homogenate in PBS. **2:** Sarcosyl solubilization. **3:** Sarcosyl extraction, pellet. **4:** Pellet obtained after sucrose gradient before PK treatment. **5:** Pellet obtained after sucrose gradient after PK treatment. **6:** 500 ng of recombinant PrP^{C}, used as a positive control**. (C)** Quantification of PrP^{RES} concentration. Known amounts of recombinant PrP^{C} were compared with different dilutions of purified PrP^{RES} by western blot analysis (left panel). The band intensity was quantified to estimate the concentration of PrP^{RES} by comparison to the values obtained with the calibration curve of recombinant PrP^{C} (right panel). R2 corresponds to the linear regression coefficient. **(D)** Neuro2a cells were treated with 1 mg/ml brain derived PrP^{RES} for indicated time points and then washed extensively with PBS. Then cells were collected by tripsinization and further washed in PBS by centrifugation. PrP levels were monitored by Western blot in total protein extracts. The molecular weight of PrP corresponds to the PK-resistant core, indicating the detection of the exogenously added brain-derived PrP. In this assay, PrP^{RES} oligomers are also observed (*). Actin levels were monitored as control.
