# Supplementary material for: Prion Protein Misfolding Affects Calcium Homeostasis and Sensitizes Cells to Endoplasmic Reticulum Stress
Source: PLoS One. 2010 Dec 29;5(12):e15658. doi: 10.1371/journal.pone.0015658 (PMC3012133; doi:10.1371/journal.pone.0015658)
Supplement: Figure S4 — Stable expression of prion mutants. Neuro2A cells were transfected withindicated PrP expressing vectors containing the 3F4 tag epitope, and then selectedwith G418 (1.3 mg/ml). The expression levels for each PrP version was assessed overtime by Western blot analysis during early selection (∼1 week, upper panel) or afterstable selection (3 week, middle panel; 4 weeks, bottom panel). As loading control anon specific band is presented from the 3F4 Western blot. Cells presented in the rightpanel were used to the viability assays. (PDF) [file pone.0015658.s004.pdf]

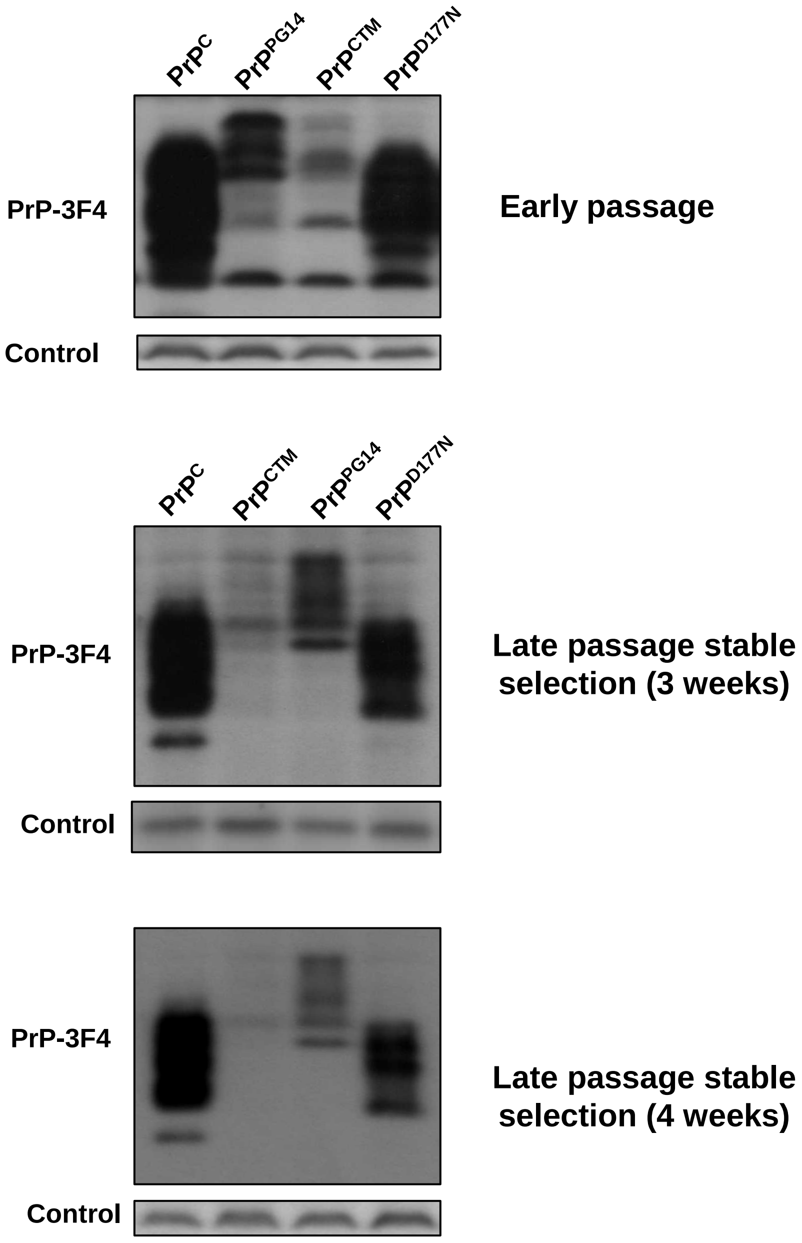

Figure S4. Stable expression of prion mutants. Neuro2A cells were transfected with indicated PrP expressing vectors containing the 3F4 tag epitope, and then selected with G418 (1.3 mg/ml). The expression levels for each PrP version was assessed over time by Western blot analysis during early selection (~1 week, upper panel) or after stable selection (3 week, middle panel; 4 weeks, bottom panel). As loading control a non specific band is presented from the 3F4 Western blot. Cells presented in the right panel were used to the viability assays.

Torres et al., Figure S4
